# SNP-Discovery by RAD-Sequencing in a Germplasm Collection of Wild and Cultivated Grapevines (*V*. *vinifera* L.)

**DOI:** 10.1371/journal.pone.0170655

**Published:** 2017-01-26

**Authors:** Annarita Marrano, Giovanni Birolo, Maria Lucia Prazzoli, Silvia Lorenzi, Giorgio Valle, Maria Stella Grando

**Affiliations:** 1 Department of Genomics and Biology of Fruit Crops, Grapevine Genetics and Breeding, Research and Innovation Centre, Fondazione Edmund Mach, San Michele all'Adige, Trentino, Italy; 2 CRIBI Biotechnology Centre, University of Padua, Padua, Italy; 3 Department of Biology, University of Padua, Padua, Italy; Universidade de Lisboa Instituto Superior de Agronomia, PORTUGAL

## Abstract

Whole-genome comparisons of *Vitis vinifera* subsp. *sativa* and *V*. *vinifera* subsp. *sylvestris* are expected to provide a better estimate of the valuable genetic diversity still present in grapevine, and help to reconstruct the evolutionary history of a major crop worldwide. To this aim, the increase of molecular marker density across the grapevine genome is fundamental. Here we describe the SNP discovery in a grapevine germplasm collection of 51 cultivars and 44 wild accessions through a novel protocol of restriction-site associated DNA (RAD) sequencing. By resequencing 1.1% of the grapevine genome at a high coverage, we recovered 34K *BamHI* unique restriction sites, of which 6.8% were absent in the ‘PN40024’ reference genome. Moreover, we identified 37,748 single nucleotide polymorphisms (SNPs), 93% of which belonged to the 19 assembled chromosomes with an average of 1.8K SNPs per chromosome. Nearly half of the SNPs fell in genic regions mostly assigned to the functional categories of metabolism and regulation, whereas some nonsynonymous variants were identified in genes related with the detection and response to environmental stimuli. SNP validation was carried-out, showing the ability of RAD-seq to accurately determine genotypes in a highly heterozygous species. To test the usefulness of our SNP panel, the main diversity statistics were evaluated, highlighting how the wild grapevine retained less genetic variability than the cultivated form. Furthermore, the analysis of Linkage Disequilibrium (LD) in the two subspecies separately revealed how the LD decays faster within the domesticated grapevine compared to its wild relative. Being the first application of RAD-seq in a diverse grapevine germplasm collection, our approach holds great promise for exploiting the genetic resources available in one of the most economically important fruit crops.

## Introduction

The introduction of molecular markers in plant breeding has enabled remarkable advances in agricultural production thanks to the discovery of genes associated to major agronomic traits, the study of species diversity and evolution, and the characterization of plant genetic resources [1]. During the last ten years, Single Nucleotide Polymorphisms (SNP) have become the most widely used markers due to their abundance in genomes. They compensate the biallelic nature by being ubiquitous and amenable to high-throughput automation [2]. The advent of Next Generation Sequencing (NGS) has increased the possibilities of *de novo* and reference SNP discovery in cost-effective and parallel manners. At the same time, huge progress has been achieved for high throughput SNP genotyping thanks to the introduction of array-based technologies, able to screen several thousand SNPs per assay [3]. SNP arrays rely on the prior production of sequence information, the identification and validation of polymorphisms and finally the array construction [4]. Myles et al. [5] designed the first SNP array for grape (Illumina Vitis9KSNP chip) by using a discovering panel of 17 genomic DNA samples from *V*. *vinifera* cultivars and wild *Vitis* species. The second high throughput SNP array (Illumina Vitis18KSNP array) was produced in grapevine as part of the GrapeReSeq Consortium [6]. Many experiments have shown how the application of these array-based technologies to population genetic studies may underestimate the real genetic diversity of the investigated populations, especially when the discovery panel is evolutionary divergent from the studied accessions [7–8].

Several methods that combine genome-wide SNP discovery and SNP genotyping are nowadays available. They rely on the use of restriction enzymes in order to reduce the portion of the genome to be sequenced. The number and type of restriction enzyme used as well as the amount of digested DNA, the multiplexing capabilities and the final depth of SNPs coverage vary between the different protocols of genome-wide SNP discovery. One of these approaches is the *Restriction-site Associated DNA sequencing* (RAD-seq) based on rare-cutter restriction enzymes (6–8 bp recognition site) for sequencing short DNA fragments surrounding a particular recognition site throughout the genome [9]. This method derives from the RAD tag marker technique [10] adapted to NGS platforms [11–12]. The RAD-seq approach produces two types of markers: a) co-dominant SNP markers within the flanking regions of the restriction enzyme site; b) dominant markers due to sequence variations of the restriction endonuclease cutting site. RAD-seq has been used in several plant species to discover SNPs, construct genetic maps and identify quantitative trait loci (QTLs) [12–13]. Recently, the RAD-seq approach has been applied to biparental populations of grape producing rather dense genetic linkage maps of around 2,000 SNPs [14–15]. Several modifications of the original RAD-seq protocol have been introduced by Genotyping-by-sequencing (GBS) [16], double digest restriction-site-associated DNA sequencing (ddRAD-seq) [17] and 2b-RAD-seq [18] methods. For instance, GBS [16] used a frequent cutter enzyme to generate reduced representation libraries prior to sequencing. GBS was first applied in grape by Barba et al. [19] to investigate the inheritance of powdery mildew (*Erysiphe necator*) resistance within a segregating population of *V*. *rupestris* x *V*. *vinifera* ‘Chardonnay’, finally mapping 35,8% of the 47K SNPs identified. Actually, one of the major drawbacks reported for GBS is the high rate of missing data which is currently faced by imputation programs such as LinKImpute [20] and Beagle [21].

The reference genome sequence of grapevine has been available since 2007 [22] with a total size of 487 Mb. Almost two million putative SNPs were reported for the heterozygous cultivar ‘Pinot Noir’ with an overall rate of 4 polymorphisms per kilobase [23]. A few other individual grapevine genomes have been completely sequenced so far. Da Silva et al. [24] analyzed the genome of the cultivar ‘Tannat’ with a mixture of *de novo* assembly and iterative mapping onto the ‘PN40024’ reference genome, identifying over two million single-base differences with the latter. At the same time, Di Genova et al. [25] by sequencing the ancient table grape ‘Sultanina’ found 1,193,566 high quality SNPs and novel genes absent in the *V*. *vinifera* ‘PN40024’ reference genome. More recently Corso et al. [26] resequenced two grape rootstocks, both interspecific hybrids, revealing a SNP frequency of one variant every 200 bases with the ‘PN40024’ reference genome. However, full genome sequences have not been published yet for the grapevine subspecies *sylvestris*, which is believed to be the wild ancestor of present cultivars [27–28]. To date, the genetic diversity within wild populations of *V*. *vinifera* as well as the genetic relationships between *sativa* and *sylvestris* genotypes have been mostly evaluated using a small number of microsatellite and SNP markers [29–30]. It has been observed that the *sativa* cultivars retain a higher level of heterozygosity than the *sylvestris* accessions [31–34]. In addition, recent studies showed low levels of Linkage Disequilibrium (LD) in *V*. *vinifera*, with a decay of LD at ~10 kb inter-SNP distances [35–37]. In particular, the LD decay appeared unchanged between *sativa* and *sylvestris* [35–36] or slower in the cultivated data set [38]. An expanded genetic scan across the genomes of *sylvestris* and *sativa* individuals would enable to perform whole-genome comparisons of the level of genetic diversity and LD between the two *V*. *vinifera* subspecies, as well as to characterize their genetic relationship and to reveal the evolutionary events occurred during the long history of viticulture [39].

In this study we describe the SNP discovery carried out for the first time in a diverse set of cultivated and wild forms of *V*. *vinifera* through a novel protocol of RAD sequencing based on the 5500 SOLiD^™^ System. The 37K identified variants were annotated in order to weight their effect by type and region, and used to assess the genetic variation within the cultivated grapevine and its wild form. This proof-of-concept study showed that the RAD-seq is able to unlock valuable genetic diversity hidden in wild relatives of grape, and to provide new patterns of the LD decay in grapevine germplasm collections.

## Methods

### Plant material and DNA extraction

A germplasm collection of 51 cultivated (*V*. *v*. spp. *sativa*) and 44 wild-type (*V*. *v*. spp. *sylvestris*) female grapevines was sorted at the FEM grape repository (ITA362), located in San Michele all'Adige, Italy (S1 Table). The *sativa* accessions were chosen within a genetic core collection (G-110) that retains 100% of SSR and SNP loci diversity present in the source collection [30]. The wild individuals, mostly originating from the Italian Peninsula, were selected within the *sylvestris* accessions of the same repository previously clustered through a hierarchical STRUCTURE analysis [30]. Young leaf tissue of one field grown plant per accession was harvested and stored immediately in sterile tubes at -80°C for DNA extraction and successive analyses. Total genomic DNA was isolated from freeze-dried tissue after grinding with the MM 300 Mixer Mill system (Retsch., Germany) using the DNeasy 96 plant mini kit (QIAGEN, Germany). DNA concentration and purity were checked both by the Synergy HT Multi-Mode Microplate Reader (BioTek) and the NanoDrop 8000 UV-Vis Spectrophotometers (Thermo Scientific).

### Choice of restriction enzyme and adapter design

RAD-seq libraries (see paragraph “Libraries construction”) were previously constructed with genomic DNA from PN40024 using three restriction enzymes (*HindIII*, *BamHI* and *NcoI*) separately that present a different number of recognition sites on the grapevine reference genome. The number of restriction sites recovered by each RAD-seq library at different coverage thresholds (number of RE site with coverage 4X, 8X, 16X, 24X; S2 Table) was checked in order to apply the best candidate RE to the entire grapevine population.

**Fig 1:**
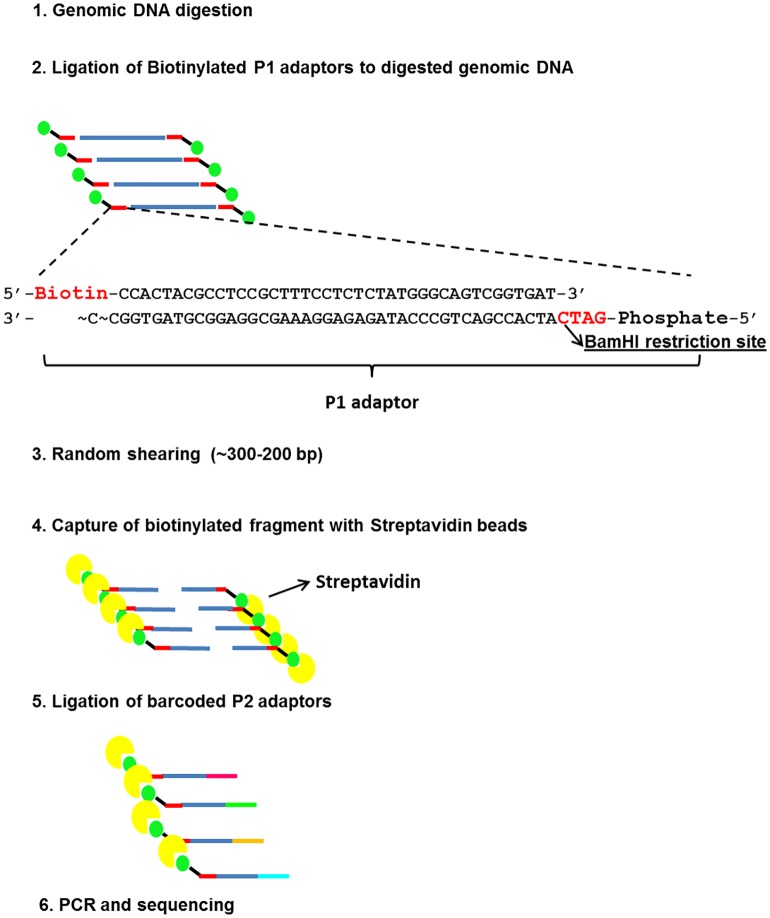
Main steps of the novel RAD-seq protocol. 1–2) sample genomic DNA is digested. The resulting digested DNA fragments are ligated to a P1 adaptor, that presents a biotin group and a 4 bp overhang complementary to BamHI recognition site. 3-4-5) Biotinilated fragments are random sheared to a target size of 300–200 bp, captured using streptavidin beads and ligated to standard barcoded adaptors for 5500 SOLiD Fragment libraries. 6) RAD-seq libraries are amplified and purified before sequencing.

Two types of adapters were used. The common 5500 Series SOLiD^™^ P1-T adapter for Fragment Library Preparation was modified by adding a biotin on the 5’ end of the top strand, and a 4 bp overhang, complementary to the sticky ends generated by *BamHI*, on the 5’ end of the bottom strand (Fig 1). The sequences of the top and bottom oligonucleotides are: 5′-Biotin-CCACTACGCCTCCGCTTTCCTCTCTATGGGCAGTCGGTGAT-3’ and 5’-Phosphate-GATCATCACCGACTGCCCATAGAGAGGAAAGCGGAGGCGTAGTGGCC-3’. The P1 adapter oligonucleotides were diluted separately in Milli-Q water (100 μM each) and then annealed in a thermocycler according to the following conditions: 95°C for 3 min, ramp down to 4°C by 1°C/30 secs; 4°C hold. The second adapter type was the standard barcoded adaptor used for 5500 SOLiD Fragment libraries and has a 10 bp barcode sequence. The different oligonucleotide sequences of the standard barcoded adapters are available on the Fragment Library Preparation 5500 Series SOLiD^™^ Systems User Guide [40]. Both biotinylated and barcoded adapters were diluted in water to 5 μM. Moreover, the presence of the restriction site in both adapters was verified in order to avoid its regeneration after the ligation with genomic DNA.

### Libraries construction

DNA samples (500 ng) were digested with *BamHI-High Fidelity* (New England Biolabs, NEB) enzyme for 1h at 37°C in 25 μL volumes containing 1X NEB CutSmart Buffer and 5U of BamHI (Fig 1). Next 30 μL of ligation master mix, containing 4 pmols of the biotinylated P1 adapter, 1X T4 DNA ligase reaction buffer (Invitrogen^™^) and 1U T4 DNA ligase (Invitrogen^™^) were added to the digestion products, and samples were incubated at 16°C overnight. The ligation products were purified using one volume of Agencourt AMPure XP beads (Beckman Coulter) according to the manufacturer’s instructions and solubilized in 50 μL of 1X Low TE (10 mM Tris-HCl, 0.1 mM EDTA). DNA fragments were random sheared with a Covaris S220 Focused-ultrasonicator in 130 μL microTUBEs AFA Fiber Snap-Cap following the manufacturer’s protocol for Target BP Peak of 200 bp. Afterwards the samples were vacuum concentrated to a final volume of 20 μL. Next 10 μL of Dynabeads^®^ MyOne^™^ Streptavidin C1 (10 μg/μL), previously washed three times with 50 μL of 2X Binding and Washing (B&W) Buffer (10 mM Tris-HCl pH 7.5; 1 mM EDTA, 2 M NaCl), were added to each sample and resuspended in 20 μL of 2X B&W. Samples were incubated for 30 min at room temperature in rotation in order to capture the biotinylated fragments. Biotinylated coated beads of each sample were separated with a magnet for 2–3 min, collecting the supernatant in a clean tube to estimate the DNA recovery rate through a Qubit^®^ 2.0 Fluorometer (dsDNA HS Assay; Life Technologies). The biotinilated coated beads were first washed with 50 μL of 1X B&W buffer and later with 50 μL of Buffer EB (Qiagen), and then resuspended in 20 μL of Buffer EB. Next 25 μL of NEBNext^®^ End Repair Module (New England Biolabs) master mix, containing 5 μl of NEBNext End Repair Reaction Buffer (10X) and 2.5 μl of NEBNext End Repair Enzyme Mix (10,000 units/ml T4 PNK; 3,000 units/ml T4 DNA Polymerase), were added to the biotinylated beads. The End Repair mix was incubated for 15 min at room temperature in rotation. After the End Repair Enzymes inactivation at 75°C for 20 min, 50 μl of ligation master mix, containing 4 pmols of the blunt barcoded P2 adapters, 1X T4 DNA ligase reaction buffer and 10U T4 DNA ligase (Invitrogen^™^), were added to the biotinylated samples and incubated 1h at room temperature in rotation. The biotinylated fragments from each library were amplified in 50 μl volumes containing 25 ng DNA fragments, 1X GoTaq^®^ Green Master Mix (Promega) and 25 pmol each of the following primers: Library PCR Primer 1, 5′ -CCACTACGCCTCCGCTTTCCTCTCTATG-3′ and Library PCR Primer 2, 5′ -CTGCCCCGGGTTCCTCATTCT-3′ [40]. The amplification was performed according to the following conditions: 95°C for 5 min, 12 cycles of 94°C for 20 secs, 62°C for 20 secs, 72°C for 50 sec, with a final Taq extension at 75°C for 3 min. PCR products were purified using 1.3 volumes of Agencourt AMPure XP beads. Each library was loaded on a Bioanalyzer (Agilent Technologies) for the evaluation of fragments size through a High Sensitivity DNA Assay. Libraries were considered suitable for sequencing if adapter dimers (99 bp in length) were minimal or absent and the majority of other DNA fragments were between 150–350 bp. If an excess of adapter dimers were present, the RAD libraries were purified again. Finally, fragments sequencing (75 bp reads) was performed on a 5500 SOLiD^™^ System (Applied Biosystems, Life Technologies) pooling the libraries and running them in two different flow-cell lanes using the Exact Call Chemistry module (ECC).

### Reads pre-processing

Reads were expected to start with the 5’-GATCC-3’ sequence released by *BamHI* cut and corresponding to T12320 in color space format. Reads 75 bp long obtained from SOLiD sequencing were inspected for the presence of the T12320 sequence at their starting point. When there were no color errors or one color sequencing error at the beginning, the read starting sequence was replaced with the full color space *BamHI* restriction site (T102320). Reads with more than one color error in their starting sequence were discarded.

### DNA sequence alignment

Pre-processed reads in color space were mapped on the reference 12X grape genome [22], the mitochondrial (mtDNA) [41] and the chloroplast (cpDNA) [42] DNA sequences using BFAST v0.7.0a aligner [43]. Only unique alignments with identity at least 90% were kept (S1 File). All statistical analysis were performed using ‘stats’ v3.4.0 [44] and ggplot2 v2.1.0 [45] R packages.

### SNP calling and annotation

The UnifiedGenotyper tool of the Genome Analysis Toolkit (GATK) v3.2–2 [46] was applied to call variants on unique alignments with a mapping quality score higher than 17. SNP genotypes were inferred through a *Bayesian* genotyper implemented in GATK that assigned genotype at each site as the genotype with the greatest posterior probability (S1 File). SNPs from different samples in regions with read depth at least 10 were then merged into a single VCF file. SNP density across the *V*. *vinifera* ‘PN40024’ reference genome was evaluated by counting the number of SNPs in sliding windows of 500 Kb using VCFtools [47]. Pearson’s correlation (r) was used to determine the relationship between the number of SNPs per chromosome and chromosome physical size. Finally, SNPs were classified into genomic feature groups and gene classes according to the grape gene annotation v2.1 [48].

### SNP validation

50 fragments were selected to validate 183 SNPs with Sanger sequencing [49]. PCR primers were designed using NCBI/Primer-BLAST [50] to yield products 266–1002 bases long (S3 Table). Target sequence fragments were amplified in 4 cultivated and 3 wild accessions chosen within the analyzed population (S4 Table). Another *V*.*v*. *sativa* variety, that showed an uncommon low level of genetic variation at microsatellite loci, was also included during Sanger sequencing in order to test the ability of RAD-seq markers to capture undisclosed genetic diversity. The products of Sanger sequencing were run on the 96-capillary 3730xl DNA analyzer (Applied Biosystems^®^). STADEN package v2.0.0 [51] was used to analyze DNA sequences. The overall rate of fitted genotypes was estimated by dividing the total number of fitted genotypes with the total number of evaluated genotypes (7*N° of confirmed SNPs).

The grapevine population investigated in this study had previously been genotyped with the commercial GrapeReseq Illumina Vitis20KSNP chip [52]. The Infinium genotyping raw data were analyzed using the Genotyping Module v1.9 of the Illumina GenomeStudio Data Analysis software [53]. An individual locus analysis, where loci are identified by sorting on per-locus metrics such as call rate and cluster separation, was carried out to obtain a final data set of good quality SNPs. The genetic profiles of the shared SNPs between GrapeReseq 20K chip and RAD-seq data sets were compared for all 94 samples in order to assess the rate of fitted genotypes between the two different approaches.

### Analysis of genetic diversity and linkage disequilibrium (LD)

Samples with a missing rate > 0.5 and SNPs with a missing rate > 0.2 were filtered out. Genotype imputation was performed to fill in the missing data using LinkImpute v1.1.1 software, which is based on a k-nearest neighbor genotype imputation method (LD-kNNi) designed to work with unordered markers [20]. Afterwards, SNPs with a minor allele frequency (MAF) lower than 0.05 were removed using Plink v1.9 software [54–55]. The final SNP panel was used to estimate the observed (*H*_*O*_) and expected (*H*_*E*_) heterozygosity, and the fixation index (*F*_*is*_, inbreeding coefficient), through the R package “diveRsity” [56]. The number of private alleles was evaluated by using the function “—freq” implemented in Plink v1.9 software [54]. Finally, linkage disequilibrium (LD) between all SNPs was estimated within *sativa* and *sylvestris* subgroups separately by using Plink v1.9 software [55]. The classical *r*^*2*^ estimate of correlation between genotypes was used [57]. LD decay was explored by plotting the median *r*^*2*^ in sequential bins of 10 Kb against physical position.

## Results

### Sequencing summary

**Fig 2:**
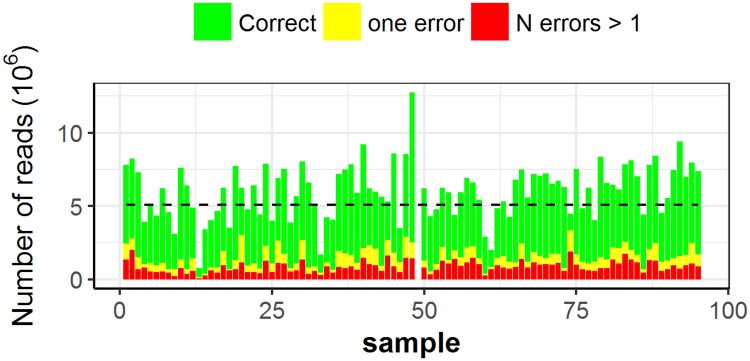
Summary of SOLiD sequencing errors at the starting sequence. Reads per sample with no colors errors (green); Reads per sample with one color error (yellow); discarded reads per sample due to color errors higher than one (red). The black dotted line indicates the average number of reads per sample.

We selected *BamHI* as candidate restriction enzyme to construct RAD-seq libraries. Indeed, it showed almost a constant and high number of recovered RE sites at different levels of coverage, compared to the other two REs used to test the technical performance of the novel RAD-seq protocol (S2 Table). RAD-seq libraries were constructed separately for 95 grapevine samples and were sequenced in two lanes using the 5500 SOLiD^™^ System. A total of 566M reads 75 bp long were produced (Table 1) with an average of 5,102,500.3 reads per sample. The coefficient of variation (CV) for the number of reads was equal to 33.9% among samples and 2.5% per sample among lanes. *BamHI* is a type II restriction endonuclease without methylation sensitivity that recognizes a six bp site (5’–GGATCC–3’), cleaving just after the first 5'-guanine on each strand. It leaves five base-long sticky ends (GATC-C) whose sequences are equal in color space format to T12320. As shown in Fig 2, 75% of the reads started with a correct T12320 sequence and 11% presented one single color mismatch that we assumed to be a sequencing error. The remaining reads (14%) showed more than one different color at the beginning sequence and were discarded. In order to increase the alignment specificity, the retained reads were pre-processed by replacing the starting sequence with the full *BamHI* restriction site in color space format (T102320), yielding finally 485M correct reads (76 bp).

**Table 1:** Number of reads and sequence produced by each filtering step during reads treatment.

Step of reads treatment	Number of reads	Sequence (Gb)
5500 SOLiD^™^ sequencing	566M	42.4
Pre-processing	485	36.8
Unique alignments	294M	22.3
Unique alignments with MapQ > 10	177M	13.4

### Alignment

**Fig 3:**
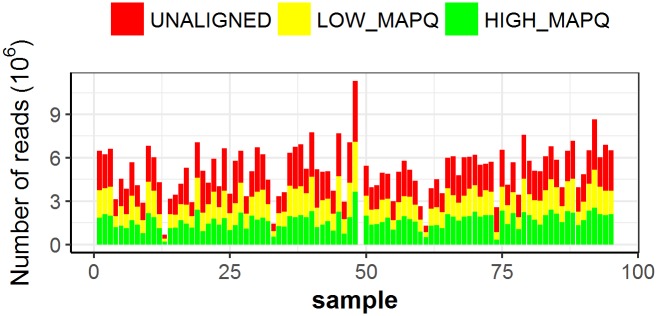
number of alignments per sample. High quality (MapQ > 10) alignments per sample are shown in green, low quality (MapQ < 10) alignments in yellow and unaligned and multiple aligned reads in red.

Pre-processed reads were aligned onto the reference 12X grape genome including mtDNA and cpDNA sequences in order to reduce the rate of multiple alignments (Fig 3). 60.3% unique alignments (Table 1) showed a mapping quality score higher than 10 (177,212,079 over 293,786,586 reads). Among them 8.4% (14,963,674) accounted for not nuclear alignments.

*In silico* digestion of the grapevine reference genome with *BamHI* identified 60,733 putative restriction sites with an average distance of 7.9 Kb. We recovered a total of 34K unique restriction sites with at least ten alignments, 93.2% of which were predicted and 6.8% were absent in the reference genome (Table 2). This sequence polymorphism rate at the recognition site may reflect the genetic variability within the investigated germplasm collection, consisting of cultivated and wild forms of grapevine. If we consider the number of recovered restriction sites, the length of a SOLiD read and the assumed presence of two reads going upstream and downstream from each restriction site (Number of covered RE *2*75bp), about 1.1% of the grapevine genome looks resequenced in our study at a high coverage in less than one hundred *sativa* and *sylvestris* accessions.

**Table 2:** Number of identified *BamHI* recognition sites.

Type of Restriction Site	Total Number
Predicted	32,080
Unpredicted	2,353
Not nuclear predicted	163
Not nuclear unpredicted	4
**TOTAL**	34,600

The RE sites found in the grapevine PN40024 reference genome through an *in silico* digestion are called “Predicted”. The RE site absent in the PN40024 genome are defined “Unpredicted”. “Not nuclear” RE sites are those identified in mitochondrial and chloroplast DNA sequences.

We considered each up- or downstream read as a RAD locus. We expected that the read depth of each RAD locus would be similar for all the sequenced RE sites if digestion and sequencing were unbiased. However, some RE sites (16.5%) showed differences in read depth between the two adjacent RAD loci. Indeed, those RE sites presented high depth (number of reads aligned to a locus > 10) in more than 80% of the samples at either upstream or downstream RAD loci. The correlation between read depth and the logarithm of restriction fragment length for 69,525 unique RAD loci covered by at least one read was very small (r = 0.08; p-value < 2.2e-16). We observed a slightly higher correlation (r = 0.12, p-value < 2.2e-16) for RAD loci from restriction fragments shorter than 10kb (71% of all unique covered RAD loci). The correlation between read depth and the logarithm of restriction fragment length was not significant (r = 0.01, p-value = 0.1458) for RAD loci coming from restriction fragments above 10kb in length (29% of all unique covered loci).

### Variant calling and annotation

**Fig 4:**
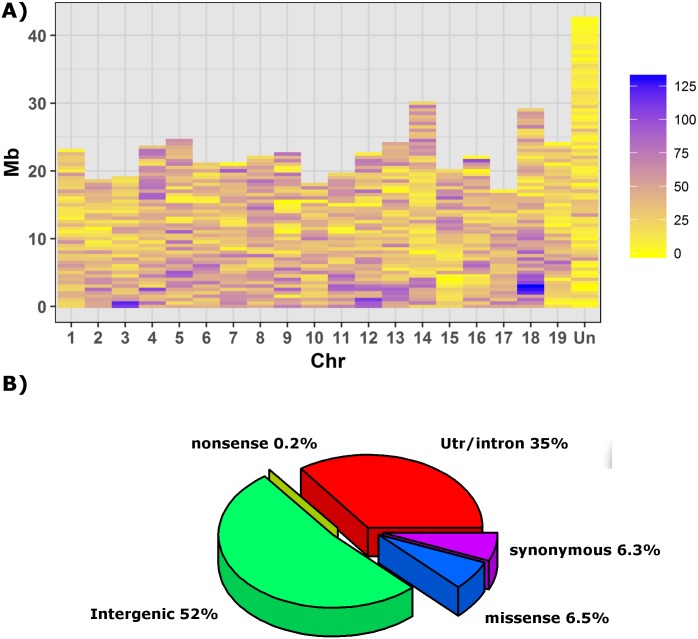
**A)** SNP density across the 12X grapevine reference genome PN40024. Each block represents a bin of 500kb. The bar “Un” shows SNPs found on unassembled genomic sequences. **B)** summary of SNPs annotation according to the grape gene annotation v2.1.

Variants on unique high quality alignments were called using UnifiedGenotyper module of Genome Analysis Toolkit (GATK) program [46]. We identified 37,748 SNPs that included 120 variants discovered on mtDNA sequence and 34 SNPs within the cpDNA genome. The 19 assembled chromosomes contained 93% of the markers with an average of 1.8K SNPs per chromosome (Fig 4A). SNP density ranged from one SNP every 10 Kb on chromosome 8 to one SNP every 16 Kb on chromosome 19. Finally, chromosome size and number of SNPs per chromosome were moderately correlated (*r* = 0.68; p-value = 0.0001). We split the reference genome in 985 bins of 500 kb and the number of SNPs per each bin was determined. Thirty-five SNPs were present on average per bin. While three bins showed zero variants, 655 bins had 10 to 50 SNPs, 83 bins had < 10 SNPs and 244 bins had 51 to 104 SNPs.

According to the grape gene annotation v2.1 more than half of the SNPs fell in intergenic regions. 18,121 SNPs belonged to 6,634 grapevine predicted genes of which 1,680 presented 2,557 nonsynonymous polymorphisms (Fig 4B). We looked for which GO terms of biological process ontology were more represented among the annotated genes showing sequence variation. An over-representation of metabolism-related functions, referring both to biosynthetic and catabolic processes, as well as of regulation and transportation mechanisms were observed. Moreover, a small but significant amount of nonsynonymous variants fell in genes related with the detection and response to stimuli such as oxidative and water stresses.

### SNP validation

Fifty PCR fragments ranging from 266 to 1,002 bp were Sanger sequenced on eight grapevine genomic DNA samples in order to validate 183 SNPs discovered by RAD-seq (S3 Table). The validation panel included four *sativa* and three *sylvestris* accessions already used to construct the RAD-seq libraries, and the Caucasian cultivar “Mgaloblishvili N.” Targeted SNPs included 123 transitions and 60 transversions, which were found at 10X coverage in at least 50 libraries. Out of 148 confirmed SNPs, 43.9% perfectly agreed with the RAD-seq data in all the resequenced samples, while 51.3% showed from one to three different genotypes (S4 Table). The overall rate of fitted genotypes was 86%, which indicates the ability of RAD-seq to determine genotypes accurately in a highly heterozygous species such as grapevine. Moreover, the exceptionally high level of homozygosity of the outer cultivated accession, that was homozygous for 49% of 312 microsatellite markers tested [58], was proved by 78% of the confirmed SNPs. Nonetheless, a heterozygous profile was still observed for 33 SNPs, highlighting how RAD-seq is able to reveal unknown genetic variability. Our RAD-seq assay sampled 115 SNPs of those included in the commercial GrapeReseq 20K chip. The last had produced a final panel of high-quality 16,563 SNPs when applied to our germplasm population. 23% of the common SNPs showed identical genotypes in all 94 samples both using the Illumina chip and the RAD-seq assays, while 72% differed in 1 to 15 cases bringing the overall rate of fitted genetic profiles among the two different genotyping approaches to 96%.

### Genetic diversity and LD decay within populations of sativa and *sylvestris* grapevines

**Fig 5:**
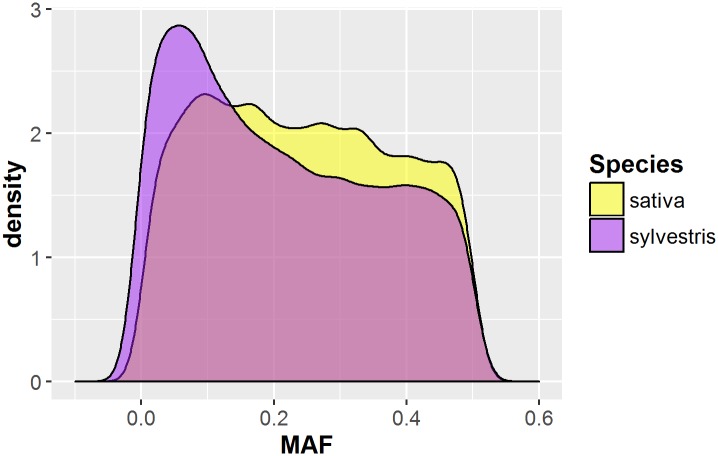
Minor allele frequency (MAF) distribution within cultivated (yellow) and wild (blue) grapevine populations, taking into account all nuclear SNP loci identified through the novel RAD-seq assay.

**Fig 6:**
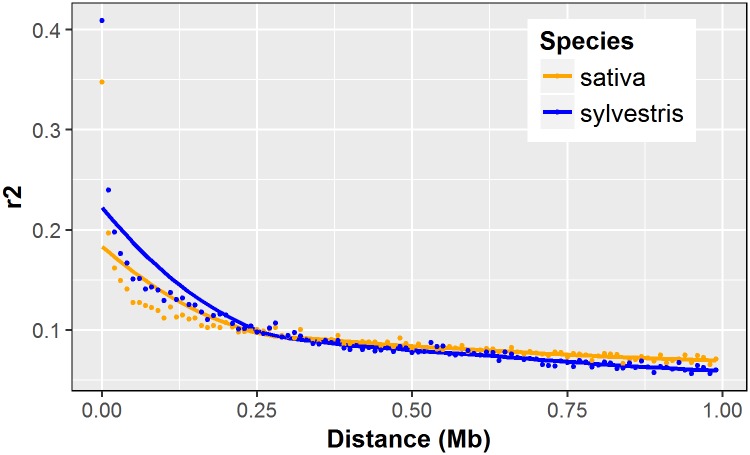
The decay of LD in *sativa* and *sylvestris* populations. Each point represents the median r^2^ value in sequential bins of 10kb against physical position.

In order to evaluate the suitability of our SNP panel for investigating the genetic relationship of wild and cultivated grapevines, we estimated the main statistics of genetic diversity in the two subspecies separately. Taking into account the nuclear polymorphisms only, we removed 21,920 SNP loci with a missing rate higher than 0.2, and 5 samples showing missing data at 50% of the markers (S1 Table). In addition, the Pinot Grigio and Pinot Meunier, two somatic variants of cv Pinot Noir, and the line Pinot Noir 40024 were not considered (S1 Table). The distribution of minor allele frequency (MAF) was quite different between wild and cultivated grapevines (Fig 5): the first showed an abundance of loci with a MAF < 0.1 (3,548 SNPs), while the latter presented a more homogenous distribution of allele frequencies. Moreover, we identified just five private alleles (PA) in the *sylvestris*, instead of the 584 PA pinpointed in the *sativa* accessions. After imputing the missing genotypes, we filtered out 1,333 markers with a MAF less than 0.05 gaining a final dataset of 14,341 SNPs. We used this final SNPs panel to assay the genetic diversity within wild and cultivated grapes. As shown in Table 3, the cultivated individuals revealed a higher level of heterozygosity compared to the wild accessions. Furthermore, the *sativa* exhibited a slightly higher value of heterozygosity (*H*_*O*_) than expected (*H*_*E*_). Finally, we tested the extent of LD in the two subspecies, carrying out a pairwise analysis between all SNPs with a MAF >5%. A slower LD decay was observed within the *sylvestris* subset, where the classical correlation coefficient r^2^ reached values below 0.2 within 20kb, compared to the *sativa* subgroup, where the LD (r^2^) decayed below 0.2 within 10 kb (Fig 6).

**Table 3:** Indices of genetic diversity evaluated in cultivated and wild accessions separately.

Population	*Sativa*	*Sylvestris*
**Size**	45	42
**H**_**O**_	0.364	0.311
**H**_**E**_	0.350	0.313
**F**_**IS**_	-0.030	0.020
**PA**	584	5

H_O_ = observed heterozygosity; H_E_ = expected heterozygosity; F_IS_ = inbreeding coefficient; PA = private alleles.

## Discussion

All the modern cultivars of grapevine belong to the species *V*. *vinifera*, one of the most important crops worldwide [59] and the only endemic taxon of the family *Vitaceae* in Eurasia and Maghreb [60]. The Eurasian grapevine exists nowadays as the cultivated (*V*. *v*. subsp *sativa*) and the wild form (*V*. *v*. subsp *sylvestris*), which is supposed to be the ancestor of present varieties [61]. The genetic relationship between the two subspecies *sativa* and *sylvestris* is still controversial [34–35]. The creation of genomic databases of reference *sativa* and *sylvestris* accessions will make it possible to characterize the relatedness between wild and cultivated grapes at genomic level and to deeply explore the natural genetic variation still preserved within wild grapevine populations [39]. In this regard, we applied a novel protocol of RAD-seq to a germplasm collection of wild and cultivated grapevine individuals. We obtained 36.8 Gb of sequences, of which over 40% did not align successfully or were mapped in multiple locations on the 12X *V*. *vinifera* reference genome (Fig 3). The same rate of unaligned reads was even observed for the sample PN40024 (Fig 3, sample 51). This may be due to the incomplete assembly of the reference genome or, with exception of the reference sample itself, to the high levels of genetic variation between the PN40024 and the investigated grapevine accessions. Similar findings have also emerged from the comparison of both “Tannat” and “Sultanina” *de-novo* assembled grapevine genomes with the reference genome [24–25]. This can be even more evident in our study since half of the population belongs to the wild Eurasian vine *sylvestris* whose genome has not been thoroughly investigated yet. By now it is well accepted that plant genomes contain core sequences that are common to all individuals, as well as dispensable sequences comprising partially shared and non-shared genes that contribute to intraspecific variation [62]. Moreover, the heterozygous cultivar Pinot Noir showed a relevant portion of hemizygous DNA that confirms how the grape genome exists in a dynamic state mediated in part by transposable elements [23]. The advances in sequencing technologies (i.e. the Single Molecule Real-Time (SMRT) Sequencing technologies) and the development of novel algorithms and software will go beyond the difficulties emerged in the assembly and alignment of grapevine genomes, as recently reported by Chin et al. [63]. Moreover, it will become possible in grapevine moving from one single reference genome to multiple reference genomes, helping to reconstruct the evolutionary history of viticulture as well as to better interpret and eventually exploit the phenotypic variation observed nowadays in natural populations [64].

More than two thousands *BamHI* restriction sites were identified in our sequences that are absent in the reference genome. The absence/presence of a restriction site could be related to loss/gain of the RE site because of mutations occurred during the grapevine evolution and propagation. The predicted restriction sites not recovered by RAD-seq assay could also be explained by imperfect digestion or poor quality reads as well as the presence of RE sites within repetitive sequences, as proved by the moderate percentage of reads discarded during the pre-processing and alignment analysis (Figs 2 and 3). A considerable level of genetic diversity within the investigated population has been proved by the 37K SNPs discovered, given that half of the investigated population is composed of wild grapevine genotypes. This panel exhibited a uniform marker density among chromosomes and significantly higher than those reported in grapevine using other Reduced Representation Library (RRL) methods [14–16]. The analysis of genetic diversity within the investigated germplasm collection supported the usefulness of the genome-wide SNP- panel developed in this study. Indeed, we observed a higher level of heterozygosity in the domesticated grapevine compared to its wild form, supporting previous observations based on few molecular markers [30, 37]. This result is clearly reflected in the excess of low frequency alleles (MAF < 0.1; Fig 5) and the low number of PA found in the *sylvestris* subset, that suggest a potential high level of inbreeding (*F*_*IS*_ = 0.020; Table 3) likely resulted from the small size of the wild populations and the absence of inter-wild populations gene-flow [65–66]. Furthermore, the lower number of PA found within the wild subgroups may indicate the purifying selection against new mutations occurring in the natural habitats of river banks, where small and isolated wild populations of grapevine can be still found [67]. On the other hand, the high genetic variability observed within the cultivated accessions may arose from sexual crossing, somatic mutations and massive vegetative propagation occurred during the grapevine evolution [61]. In addition, the analysis of genetic diversity revealed a slightly higher H_O_ than H_E_ in the *sativa* subset, that might indicate an excess of outbreeding as well as events of migration or balancing selection [68]. These results are related with the ability of RAD-seq methods to identify and score markers simultaneously in the investigated population, surpassing one of the major limitation of SNP array technologies, that are often based on the genetic diversity discovered in a few resequenced individuals. For instance, the Vitis20K chip comprises 18,071 SNPs discovered within 47 *V*. *vinifera* genotypes and other 18 *Vitis* species [6]. Out of the *V*. *vinifera* genotypes just four accessions are *sylvestris*, which likely leads to an underestimation of the genetic diversity in wild grape populations. Instead, our grapevine germplasm collection included 44 wild grapes, whose authenticity has been assessed by combining ampelometric [69] and molecular analysis [30]. The simultaneous discovery and genotyping of SNPs in a RAD-seq assay can also increase the number of high-quality markers useful in further analysis. In this regards, we measured the extent of LD in the two subspecies separately, revealing how the LD decays slowly in the wild form compared to the domesticated grapevine. This result contrasts with previous reports on LD decay between *sativa* and *sylvestris*, where it appeared unchanged among the two subspecies [35–36] or slower in the cultivated data set [38]. This discrepancy is not surprising since in general LD extent can vary according to different factors, such as the population under investigation, its mating system and history, as well as the occurrence of natural and artificial selection [70]. In our case the longer extent of LD observed in the wild accessions can be related with an elevated level of inbreeding, already suggested by the above analysis of genetic diversity [67, 29]. The common geographical origin of most *sylvestris* could also explain the slower LD decay as well as the low level of genetic diversity observed (S1 Table). However, a previous survey [71] based on the distribution of chlorotypes within populations of wild grapevines collected across the Mediterranean basin has shown the highest within-population diversity in the Italian Peninsula, which may be proposed as a refugia of wild populations. Nevertheless, our results highlights that the SNP genome coverage and the molecular diversity captured by our RAD-seq assay had a significant contribution towards shaping the LD patterns in the wild and cultivated grapevines [72].

A further evidence of the high level of heterozygosity in grapevine plants was the high number of variants found in less than 1 Mb [73]. This high genetic variability can be challenging for genome-wide polymorphisms discovery and genotyping [74]. In RRL approaches restriction site heterozygosity can skew read depth, leading to discarding low coverage RE sites, and it can cause null alleles at flanking SNP loci [75]. Since this bias depends on the size of the sample assayed and on the level of restriction site conservation across the sample, more individuals are sequenced, a larger fraction of variants will be identified. Indeed, sequencing many individuals at low depth has a higher rate of polymorphisms discovery and fair accuracy in genotype inference compared to high coverage sequencing for a few individuals [76]. Our effective sequencing coverage—1.1% of the genome in 95 wild and cultivated genotypes—has permitted finding about 2% of the expected polymorphisms based on the SNP frequency in whole-sequenced grapevine varieties [24–25]. Low coverage sequencing may soften the bias of restriction fragment length on RAD loci read depth. Indeed, Davey et al. [77] reported a correlation between restriction fragments length and read depth of RAD loci, which could be related to the shearing step during RAD library preparation, regardless of the shearing technique applied. We found that the bias was significantly lower, or almost absent, compared to Davey et al. [77] for RAD loci from restriction fragments below 10Kb. Therefore, a lower distortion of RAD loci read depth, with special regard to those up- and downstream of a heterozygous restriction site, might be expected in our RAD-seq assay.

Given that the coding regions are about 46% of the grapevine genome [23, 48], an interesting result of our study is that 48% of the identified SNPs fell in genic regions, of which the annotated ones are mostly assigned to the functional categories of metabolism and regulation. Actually, plant metabolism is the most represented functional category among the unique set of predicted genes in the grapevine genome [78]. On the other hand, the polymorphisms observed in genes related to both biosynthetic and catabolic processes as well as regulatory or transport functions may reflect different adaptation mechanism among wild and cultivated grapevines. The identification of sequence polymorphisms within genomic regions associated to metabolism and regulation pathways makes our SNP panel rather informative for discovering the genetic mechanisms that contribute to the phenotypic variation associated with domestication traits. It may be exploited in further surveys to select candidate polymorphisms contributing to domestication-related traits and to investigate the molecular pathways associated with plant response to environmental stimuli. In addition, our findings sets the stage for further applications of population genetics methods to capture the signals of selection left during the weak domestication process of grapevine and to access the unexplored genetic diversity of wild grapevine individuals [79].

## Supporting Information

S1 Table List of the grapevine accessions included in the SNP discovery panel. 'True-to-type' variaties are marked in bold. Samples excluded from the analyses of genetic diversity and LD are marked with an asterisk.
(XLSX)

S2 Table Number of restriction sites recovered with RAD-Seq on PN40024 genomic DNA using three different restiction enzymes. (XLSX)

S3 Table List of primers used for the SNP validation with Sanger sequencing. The SNPs included in the PCR-products are indicated between squared brackets.
(XLSX)

S4 Table RADseq genetic profiles of the validation panel at the 148 SNPs confirmed with Sanger sequencing. Unfitted genotypes between RAD and Sanger assays are marked in red, with the Sanger genetic profile indicated in brackets. Missing genotypes are represented as “0 0”.
(XLSX)

S1 File Scripts used for alignment and variant calling. (PDF)
