# Supplementary material for: SNP-Discovery by RAD-Sequencing in a Germplasm Collection of Wild and Cultivated Grapevines (V. vinifera L.)
Source: PLoS One. 2017 Jan 26;12(1):e0170655. doi: 10.1371/journal.pone.0170655 (PMC5268455; doi:10.1371/journal.pone.0170655)
Supplement: S1 File — (PDF) [file pone.0170655.s005.pdf]

#BFAST INDEXING

```
bfast fasta2brg -f vvinifera_all.fasta -A 1
I=0
for M in 1111111111111111111111 111110100111110011111111111 10111111011001100011111000111111
1111111100101111000001100011111011 111111110001111110011111111
11111011010011000011000110011111111 1111111111110011101111111 111011000011111111001111011111
1110110001011010011100101111101111 111111001000110001011100110001100011111
do
bfast index -n 20 -f vvinifera_all.fasta -A 1 -m $M  -w 14 -i $I
I=$((I + 1))
done
```

READ PREPROCESSING

```
zcat sample.csfastq.gz | sed "s/^!//" > sample.bfast.csfastq
```

BFAST ALIGNMENT

```
bfast match -n 20 -f vvinifera_all.fasta -A 1 -r sample.bfast.csfastq -K 100 -M 500 > sample.bmf
bfast localalign -n 20 -f vvinifera_all.fasta -A 1 -m sample.bmf -o 10 > sample.baf
bfast postprocess -n 20 -f vvinifera_all.fasta -A 1 -i sample.baf -a 3 > sample.sam
```

INDEL REALIGN

```
gatk -R vvinifera_all.fasta -I sample.bam -rf BadCigar --allow_potentially_misencoded_quality_scores -T LeftAlignIndels
-o sample.re.bam
```

#VARIANT CALLING

```
gatk -R vvinifera_all.fasta -I sample.re.bam -rf BadCigar --allow_potentially_misencoded_quality_scores -T
UnifiedGenotyper -o sample.raw.vcf --genotype_likelihoods_model BOTH -nt 20
```
